# Resident and family perceptions of the nurse practitioner role in long term care settings: a qualitative descriptive study

**DOI:** 10.1186/1472-6955-12-24

**Published:** 2013-09-27

**Authors:** Jenny Ploeg, Sharon Kaasalainen, Carrie McAiney, Ruth Martin-Misener, Faith Donald, Abigail Wickson-Griffiths, Nancy Carter, Esther Sangster-Gormley, Lori Schindel Martin, Kevin Brazil, Alan Taniguchi

**Affiliations:** 1 School of Nursing, McMaster University, 1280 Main Street West, Hamilton, ON L8S 4K1, Canada; 2 Department of Health, Aging and Society, McMaster University, 1280 Main Street West, Hamilton, ON L8S 4K1, Canada; 3 Department of Psychiatry & Behavioural Neurosciences, McMaster University, 1280 Main Street West, Hamilton, ON L8S 4K1, Canada; 4 Geriatric Psychiatry Service, St. Joseph’s Healthcare, 100 West 5th Street, Hamilton, ON L8N 3K7, Canada; 5 Dalhousie University, School of Nursing, Box 15000, 5869 University Avenue, Halifax, NS B3H 4R2, Canada; 6 Canadian Centre for Advanced Practice Nursing Research, McMaster University, 1280 Main Street West, Hamilton, ON L8S 4K1, Canada; 7 Daphne Cockwell School of Nursing, Ryerson University, 350 Victoria Street, Toronto, ON M5B 2K3, Canada; 8 University of Victoria School of Nursing, 3800 Finnerty Road, PO Box 1700 STN CSC, Victoria, BC V8W 2Y2, Canada; 9 School of Nursing and Midwifery, Queen’s University Belfast, 97 Lisburn Road, Belfast BT9 7BL, Northern Ireland; 10 Department of Family Medicine, McMaster University, McMaster Innovation Park 175 Longwood Road South, Suite 201A, Hamilton, ON L8P 0A1, Canada

**Keywords:** Nurse practitioner, Long term care, Qualitative descriptive, Perceptions, Residents, Family members, Person-centred care

## Abstract

**Background:**

Research evidence supports the positive impact on resident outcomes of nurse practitioners (NPs) working in long term care (LTC) homes. There are few studies that report the perceptions of residents and family members about the role of the NP in these settings. The purpose of this study was to explore the perceptions of residents and family members regarding the role of the NP in LTC homes.

**Methods:**

The study applied a qualitative descriptive approach. In-depth individual and focus group interviews were conducted with 35 residents and family members from four LTC settings that employed a NP. Conventional content analysis was used to identify themes and sub-themes.

**Results:**

Two major themes were identified: NPs were seen as providing resident and family-centred care and as providing enhanced quality of care. NPs established caring relationships with residents and families, providing both informational and emotional support, as well as facilitating their participation in decision making. Residents and families perceived the NP as improving availability and timeliness of care and helping to prevent unnecessary hospitalization.

**Conclusions:**

The perceptions of residents and family members of the NP role in LTC are consistent with the concepts of person-centred and relationship-centred care. The relationships NPs develop with residents and families are a central means through which enhanced quality of care occurs. Given the limited use of NPs in LTC settings, there is an opportunity for health care policy and decision makers to address service inadequacies through strategic deployment of NPs in LTC settings. NPs can use their expert knowledge and skill to assist residents and families to make informed choices regarding their health care and maintain a positive care experience.

## Background

Nurse practitioners (NPs) have provided services in some long term care (LTC) homes in the Unites States since the 1970s [1], in Canada since 2000 [2,3], and only recently in the United Kingdom [4]. This trend has helped address critical issues in LTC homes such as the increasing proportion of older residents with complex health problems, limited physician services, inadequate quality of care, and escalating healthcare costs [3,4]. While definitions of advanced nursing practice vary internationally, in Canada it is an umbrella term used for an “advanced level of clinical nursing practice that maximizes the use of graduate educational preparation, in-depth nursing knowledge and expertise in meeting the health needs of individuals, families, groups, communities and populations” (p41) [5]. The two advanced nursing practice roles recognized in Canada are the NP and the clinical nurse specialist (CNS). The NP, in comparison to the CNS or the Registered Nurse, has an expanded scope of practice and is able to autonomously diagnose, order and interpret diagnostic tests, prescribe pharmaceuticals and perform specific procedures within the legislated scope of practice [5]. Very few LTC homes in Canada employ an NP; for example, in Ontario’s 634 LTC homes there are only 13 Full Time Equivalent NPs [6]. Most homes are staffed primarily by minimally trained healthcare aides, supported by a smaller proportion of Registered Practical Nurses and even fewer Registered Nurses [7].

There is now strong research evidence to support the positive impact of Advanced Practice Nurses working in LTC settings [8]. Positive outcomes include: (a) greater improvement or reduced rate of decline in incontinence, pressure ulcers, aggressive behavior and loss of affect in cognitively impaired residents [9], (b) lower use of restraints with no increase in staffing, psychoactive drug utilization or serious fall-related injuries [10], (c) improved or slower decline in some health status indicators including depression [11], and (d) improvements in meeting personal goals [12]. There is also evidence that use of NPs in LTC settings results in lower hospitalization rates and hospital costs [13,14], lower emergency department and acute care service costs [15,16], and improved access to care [17].

The shift in health care systems towards improved patient- and person-centred approaches [18,19] suggests that resident and family perceptions of NP care in LTC are also important outcomes to consider. Person-centred practice has been defined in various ways, with common elements including trusting relationships, sharing knowledge, and respecting a person’s right to make their own decisions [20-22]. There has been some research on the use of person-centred approaches in health care including LTC settings [23]. Indeed, there have been a number of initiatives related to ‘Putting Patients First’ in healthcare [24] including the widely recognized work of the Picker Institute [25,26]. However, a gap exists in understanding the perceptions of LTC residents and family members related to the role of the NP in LTC.

While studies have examined the perceptions of staff, physicians, administrators and directors of care related to the NP role in LTC settings [3,27-29], few have explored the perceptions of residents and family members. A few quantitative studies have examined resident and family satisfaction with care provided by NPs in LTC homes. Garrard and colleagues evaluated the impact of geriatric nurse practitioners (GNPs) employed by nursing homes on quality of patient care and residents’ outcomes [30]. They found no significant difference in residents’ satisfaction with care provided by staff and the home environment.

The EverCare project has used NPs to provide primary care oversight and coordination to residents in nursing homes for many years [31]. A quasi-experimental study found that in comparison with non-EverCare controls, family members in the EverCare sample were more satisfied with the resident being seen often enough to treat problems, and the NP or physician spending enough time with the resident and explaining health care problems so they could be understood [32].

Liu and colleagues surveyed family members’ satisfaction with care provided by NPs to nursing home residents with dementia at end-of-life [33]. Overall satisfaction was significantly associated with NP-family communication and the NP providing comfort for the resident. Qualitative comments suggested that respondents valued having an ongoing relationship with the NP and that they were satisfied with being kept informed of changes in residents’ status. However, the authors noted that having a single statement inviting qualitative comments was a study limitation and recommended qualitative research with in-depth interviews be conducted. Finally, in an evaluation of the introduction of an NP to a Canadian nursing home, Klaasen and colleagues found that family satisfaction with the quality of health care improved by 24% but found no change in resident satisfaction [16].

In summary, little is known about resident and family perceptions of the NP role in LTC. Existing research has primarily used quantitative approaches to focus specifically on satisfaction with care. We could find no research that uses a qualitative, in-depth approach to more fully understand resident and family perceptions of the NP role in LTC. Such research is necessary not only to improve our understanding of resident and family perceptions of the NP role, but also to shape improvements in person-centred care and high quality care in LTC settings. Research findings will have importance for education and training of NPs to support high quality person-centred care in LTC settings and in broader policy and decision making related to allocation of NPs to LTC homes.

### Study purpose

In this paper, we report data obtained from a larger two-phase sequential mixed methods study [34] examining the integration of the NP role in Canadian LTC settings [35]. Phase One involved a survey of NPs working in LTC in Canada and their administrators and Nursing Directors of Care. Phase Two was a qualitative study of four LTC settings where NPs provided care, and included interviews and focus groups with residents and family members, health care providers, administrators, and Nursing Directors of Care. The aim of this paper is to explore the perceptions of residents and family members regarding the role of the NP in LTC settings (data collected in Phase Two of the larger study).

## Methods

### Design

A qualitative descriptive approach was used [36]. This design provides a comprehensive summary of a phenomenon in everyday language and is ideal when direct descriptions of the phenomenon are desired. This qualitative design was the ideal approach for the study aim.

### Setting

Purposive maximum variation sampling [37] was used to identify four LTC settings that represented diversity in characteristics including: (a) funding model for the LTC setting, (b) funding source for NP role, (c) setting location (urban/rural), and (d) geographic area of Canada. (See Table 1).

**Table 1:** Description of settings

**Characteristics**	**Setting one**	**Setting two**	**Setting three**	**Setting four**
Funding model for LTC setting-	For-profit	Not-for-profit	For-profit	Not-for-profit
Funding source for NP role	Government	Mixed government/ LTC setting	LTC setting	Mixed government/LTC setting
Location	Rural/suburban	Urban	Suburban	Urban
Number of sites in setting	Multiple	Single	Single	Single
Setting’s bed capacity	200 plus	200 plus	200 plus	200 plus
Focus of NP role	Direct clinical care for residents; collaboration, consultation and referral; teaching and coaching; communication and counseling; leadership	Direct clinical care for residents; collaboration, consultation and referral; teaching and coaching; communication and counseling; leadership; research	Direct clinical care for residents; collaboration, consultation and referral; teaching and coaching; communication and counseling	Direct clinical care for residents; collaboration, consultation and referral; teaching and coaching; communication and counseling
Years NP in position	> 5 years	2-5 years	> 5 years	< 2 years
Average number of hours NP on site/week	39 (between all sites)	40	37.5	40
Average number of resident contacts/week by NP	108 (average for all sites)	45	120	40

### Participants

Residents and family members in the four LTC settings were initially identified and contacted by LTC home personnel (e.g., NPs, Nursing Directors of Care, and administrative staff) who provided them with a study information letter. Those who expressed an interest in the study were met by a Research Assistant or research team member who obtained informed consent prior to the interview or focus group discussion. Participants were able to speak English. Participants were offered a $50 honorarium, in recognition of their time and travel costs. Residents, aged 60 and over, included nine females and five males. Family member participants included 15 females and 6 males (See Table 2 for summary of participants by setting). Family members were aged 40 and over, and included spouses and children of residents.

**Table 2:** Summary of participants by setting

**Characteristics**	**Setting one**	**Setting two**	**Setting three**	**Setting four**
**Type of resident interview**	1 Focus group	1 Focus group	1 Focus group	1 Focus group
Number of resident participants	2	3	6	3
Gender of residents (Female/Male)	2/0	3/0	3/3	1/2
**Type of family member interview**	3 Individual interviews	1 Focus Group	1 Focus Group	1 Focus Group
Number of family member participants	3	4	8	6
Gender of family members (Female/Male)	2/1	3/1	7/1	3/3

### Data collection

Data were collected from residents and family members from October to December 2010 using a semi-structured interview guide. Researchers conducted four focus groups with residents, three focus groups with family members, and three individual interviews with family members. Focus groups and interviews were used as they were conducive to gaining understanding of the experiences and perceptions of participants related to the NP role. Focus groups facilitated the opportunity to observe group dynamics in discussions related to NP roles. Focus groups and interviews were conducted by three PhD-prepared co-investigators and two Masters-prepared research assistants, each with experience in conducting qualitative research including focus groups and interviews. Each focus group was co-facilitated by a co-investigator and research assistant and training was conducted to ensure consistency in approach across the facilitators. Focus groups and interviews took an average of 30–60 minutes. Participants were asked to share their perceptions about the NP role in the home, their experiences with the NP, their comfort and satisfaction with the NP, the benefits of the NP role and what could be improved (interview guide available on request). Due to the research team members’ availability for travel, data collection was planned to occur over a two to three day period at each case site. During this time we aimed to conduct separate focus group interviews with approximately six residents and family members per site. While the long-term care home personnel made efforts to recruit the desired number of participants, family members’ interest and availability as well as residents’ cognitive ability oftentimes limited their participation in the study. Due to these constraints, data collection was stopped at the conclusion of the planned data collection period at each case site.

### Data analysis

Data from the interviews and focus groups were transcribed verbatim and analyzed using NVivo 9.0 software [38]. Important concepts identified from the data were labeled, coded and categorized using conventional content analysis methods [39]. Three research team members (JP, SK, CM) led the data analysis and made final decisions on the interpretation of the findings. They independently coded three transcripts, then discussed a preliminary coding framework. For focus group data, group interaction data was analyzed separately and integrated into findings with other data [40]. The coding framework was revised in a research team meeting with six additional investigators, then used to independently code the remaining transcripts. Codes were categorized and themes and sub-themes were identified. Team teleconferences were used to discuss and refine the analytic framework.

A number of strategies were used to promote qualitative rigor, specifically credibility, dependability, confirmability and transferability of findings [41]. To promote credibility, triangulation of data sources and investigator triangulation were used. Triangulation of data sources involved the inclusion of four distinct sites from across Canada, residents and family members, and interviews and focus groups. In terms of investigator triangulation, data were collected by five of the study investigators, which helped to ensure that they remained ‘close to the data’ during analysis and that their different perspectives were brought to bear on the analysis itself. Results of data analyses led by three research team members were reviewed and discussed by six additional research team members, who confirmed interpretation of most findings and refined others, which enabled data to be interpreted in a nonbiased manner. We were careful to fairly represent the perceptions of selected residents in one focus group who expressed more negative views of the NP role, in particular related to her attempts to prevent hospitalization of residents.

Dependability was promoted through the provision of an in-depth description of the study methods. Study confirmability was promoted through the use of an audit trail including notes on analytic decisions, investigator triangulation, and member checking. A form of member checking was completed during the focus groups and interviews. Facilitators took summary notes of key ideas and shared them with participants at the end of the interviews. For example, facilitators made summary statements such as “I understand that the NP helped you with a, b and c…” Participants were asked to provide feedback and clarification on the facilitators’ interpretations of the findings. Finally, transferability was promoted through a thick description of the settings, sample and methods, as well as the inclusion of four distinct settings.

### Ethical considerations

Ethics approval was granted by five Canadian university ethics boards: Ryerson University, McMaster University, University of Waterloo, University of Victoria and Dalhousie University; in addition, we obtained approval from the appropriate provincial/regional health authorities and/or long-term care homes’ ethics or quality assurance committees when necessary. Researchers ensured voluntary participation, informed consent and protection of confidentiality. At the start of the interviews or focus groups, all participants received information about the study purpose, signed consent forms, and were informed they could withdraw at any time or refuse to answer any questions that made them feel uncomfortable. Participants were told that their individual comments would remain confidential, to encourage them to be honest about their perceptions of the NP role. Due to the small number of LTC homes with a full-time NP, there was potential for participant identification, so a limited description of the settings and participants were included to protect confidentiality.

## Results

Two major themes describe the perceptions of residents and family members of the NP role in LTC settings: (a) providing resident and family-centred care, and (b) providing enhanced quality of care (See Table 3). Each theme and relevant sub-themes are described in the following sections with illustrative quotes identified by setting (e.g., S1 refers to Setting 1), interview (INT) or focus group (FG), and resident (RES), family member (FM) or Facilitator (FAC) identifier.

**Table 3:** Resident and family perceptions of the nurse practitioner role: themes and sub-themes

**Theme**	**Sub-theme**
Providing resident and family-centred care	Establishing a caring relationship Knowing the resident Providing informational and emotional support Facilitating participation in decision making
Providing enhanced quality of care	Improving availability and responsiveness to meet resident/family needs Ensuring more timely access to care Preventing unnecessary hospitalization Fostering professional working relationships

### Providing resident and family-centred care

The first major theme identified was that participants perceived the NP as providing resident and family-centred care. This theme, composed of four subthemes, reflects participants’ views that the NP established a caring and respectful relationship with them and that she had an intimate knowledge of the resident which she used to provide person-centred care. Further, participants viewed the NP as providing both informational and emotional support, regularly informing them of changes in the resident’s health and health care and actively involving them in care decisions.

### Establishing a caring relationship

Residents and family members described the foundational role of the NP in establishing a caring relationship with them. They described this relationship with terms such as “she really cares”, “compassionate”, “has a heart”, and “treats you with respect.”

RES 1: But NP has a heart. A very big one. And that is what…

RES 2: That’s what we need. We are not a number, we are not a room number….

RES 1: Yes. She really cares. (S2 FG RES)

Participants described how they felt “comfortable” and “relaxed” with the NP. They stated that their relationship with the NP put their minds at ease. They explained the stress of having a family member in a LTC home and the sense of security they had knowing the NP would take care of issues as they arose.

What has been good is that she [NP] does really care and she’s not just talking down to you either…She came and talked to me…Because it’s a difficult situation when your parent is in a place…So then I don’t have to worry. (S1 INT FM)

The above quotes reflect the sense of being treated as a person (and not a number) and with respect. In a few cases, participants described a reciprocal relationship where not only could they share their emotions with the NP, but that the NP also shared her emotions with them. One participant described how she and a NP supported each other after her mother died, concluding:

NP came in and held my hand and cried with me. And that is how fabulous that woman is. (S2 FG FM)

### Knowing the resident

Participants described the importance of the NP knowing the resident as an aspect of providing resident and family-centred care. These descriptions were focused primarily on health status such as an intimate knowledge of often complex health conditions and noticing “subtle changes” in health status.

She just came around and…said “I’m here to help you…with your diabetes and different things.” I like her, she’s very knowledgeable [about my condition]. (S4 FG RES)

For the first time since mother has been institutionalized, we feel like there is somebody [NP] with the requisite skills who knows her and is there….we didn’t have a sense that there was somebody at that level that knew her or knew her circumstance…now we feel differently. (S4 FG FM)

Participants explained that this knowing of the resident resulted in earlier diagnosis and more rapid responses to health issues. They recognized that because NPs were in the home more regularly than the physicians, NPs had a deeper understanding of residents.

Actually the NP probably knows more about my mom’s case than the doctor does…. My mom has a very complicated health picture and she knows everything off the top of her head about what has been going on with my mom. (S1 INT FM)

Further, NPs were seen to be very effective in sharing their in-depth knowledge of the resident with specialists and hospital professionals.

### Providing informational and emotional support

The third sub-theme involved the NP role in providing informational and emotional support. Participants spoke extensively about how the NP provided informational support, using terms such as “informing,” “updating,” “explaining,” “passing on information,” and “contacting.” One resident described the NP support during a time when they were confined to their room due to an infection as follows:

NP came in every day and sat and talked to me…she boosted my spirit up. She said “I know it’s terrible to be shut in your room. If you can just stick it out.” (S1 FG RES)

Participants talked about the important NP role of keeping family informed about changes in the resident’s health status or medication.

I know she’s talked to my family. She’s phoned my daughter and told her exactly what medication I am going on and talked to her about everything. (S1 FG RES)

Family members emphasized the importance of being kept up to date on the health status of the resident and changes in care provision. They valued the NP taking the initiative to contact them promptly when there were changes, as well as on a regular basis. Participants described sharing of information as a “*two-way street*” where there was a “*lot of communication back and forth*” between the NP and residents and family members.

Residents and family members described the NP as providing emotional support, “someone who takes time to really listen,” in comparison with physicians who were perceived as too busy. One participant explained that the provision of informational support also constituted emotional support when family lived some distance from the LTC home:

Living two hours away, it was a big worry. I can get back and forth as much as I could but I knew that I could count on that NP to pick up the phone and let me know what was going on with my mother. (S2 FG FM)

A family member eloquently and powerfully described the NP role as a midwife or doula. A doula is a nonprofessional who assists women and their family members before, during or after childbirth, by giving information, emotional and physical support:

She [NP] helps me and my sister a lot just by listening and providing suggestions…. Not just communicating but she is also listening. It’s almost like having a midwife or a doula or something like that, from an emotional point of view. (S1 INT FM)

### Facilitating participation in decision making

Family members and residents described the vital role of the NP in facilitating their participation in decision-making. Residents emphasized the importance of simply being included:

FAC: Anything else [that the NP does to help you]?

RES: Just to be including me.

FAC: Including you?

RES: Yes.

FAC: Okay, that sounds like that was really important for you.

RES: It’s important to all of us. (S4 FG RES)

Another resident describe how the NP included them in decisions related to their care:

FAC: Anything in the last couple of weeks or months that she’s helped you with?

RES: Coming down and talking about this pending meeting…she clued me in....I’d be blessed that they include me in decisions that…

FAC: That related to you. Those decisions related to your care, right?

RES: Yes. (S4 FG RES)

Family members described how they appreciated the NP including them in decisions related to resident care:

If there were issues where decisions had to be made, whether certain kinds of care were to be undertaken, she would call me no matter what time of day or night. I always felt that was a service so valuable to family members who weren’t right here on the scene. (S2 FG FM)

Family members spoke about the NP facilitating their participation in end-of-life decision making. One family member described feeling supported and confident in having the NP “present” and participating in the decision making process during this challenging time:

The ultimate thing that I appreciated most about having the NP here was at the time of my mother’s apparent about-to-die situation where we sat together and made the decision about whether or not to continue treatment. I felt such support and confidence in having her perspective…The NP really was tremendously helpful and very, very present with me at that time. (S2 FG FM)

### Providing enhanced quality of care

The second major theme identified from participant data was the perceived role of the NP in providing enhanced quality of care. This theme, composed of four sub-themes, reflected perceptions that care by an NP resulted in improved availability and timeliness of care, prevention of unnecessary hospitalization and more effective professional working relationships. For residents and family members, these aspects of enhanced quality care addressed healthcare problems they had experienced in LTC settings.

### Improving availability of care and responsiveness to resident/family needs

Participants identified the NP role as improving availability of care and responsiveness to resident and family needs. Participants frequently described the NP as being “available,” “accessible,” “easy to contact or connect with” and having an “open door.” Participants described how the NP encouraged them to contact her with any concerns. They felt the NP was more readily available to address care needs, including complex care needs, than the physician:

It’s so important because if it’s a weekend or whatever it is, NP is there…She’ll come…whatever time of day it is, she comes. (S2 FG RES)

She took on the job of trying to analyze what was going on with mom and she ran a series of tests and send it out to the hospital, and we had x-rays and things done, there was follow through on everything. (S4 FG FM)

### Ensuring more timely access to care

The second sub-theme is that the NP is perceived to ensure more timely access to care. Timely access to care was important to residents and family members given the limited physician availability in LTC settings. Two residents described how the NP improved their access to care as follows:

RES 1: And where I couldn’t get care, she has really helped me to tremendously… She helped with the skin cancer, the bad legs…when I couldn’t get anyone to come when I had pneumonia before they called NP and she certainly took right over and got me back on my feet and she did it again this time and I am indeed grateful to her…

RES 2: She [NP] is a godsend. I think I would have lost my leg or my foot. And my doctor wasn’t around…very hard to contact…but she was there… Available not just for me, for an awful lot of people. (S2 FG RES)

Two family members explained that NPs “speed the system up” by taking on activities usually done by physicians.

I like the immediacy of it. That it was right away and she was right on top of it. And I really appreciated the way that she was with mom….and getting things happening. (S4 FG FM)

The NP is on site and has the ability to prescribe. If my mother had to wait for the Digoxin to be prescribed by a doctor who is goodness knows where, she would have been goners long since. (S2 FG FM)

Family members emphasized the importance of having timely communication with the NP about resident health issues.

She [NP] will call us right back and explain everything to us…If there’s something we didn’t know, she would tell us…phone us right away, immediately, and fill us in. (S3 FG FM)

Participants also described the NP role in ensuring timely access to tests and specialists:

I couldn’t believe how fast my mom got to see three specialists…So with the NP, she helped make the arrangements at the hospital side. And that is a lot of caring and a lot of knowing who to talk to. (S1 INT FM)

### Preventing unnecessary hospitalization

The next sub-theme was the NP role in preventing unnecessary hospitalization. Family members described previous experiences where residents were sent to hospital for tests or procedures and had to wait for extended periods of time for care, sometimes returning to the LTC home in the middle of the night, exhausted. Wait times created special difficulties for residents with dementia. Participants viewed the NP role in avoiding emergency department wait times as enhancing resident quality of life. A family member whose 95-year old parent spent 15 hours in the hospital explained:

To me, it would be worth extra money to go to a place [LTC home] that had somebody [NP] who could do that sort of thing rather than go some place where if your loved one happens to end up in a hospital they could be there for…15 hours. (S1 INT FM)

In one setting, the NP facilitated the establishment of a videoconferencing system between the LTC home and the hospital. Residents recognized the value of this service as it meant they did not have to go to the hospital and experience tiring travel, the expense, and wait times for care.

They set up a program where they can televise me here and read it in the hospital….NP had a lot to do with having this set up….it means you don’t have to travel and maybe wait an hour or two, three hours in the hospital. (S1 FG RES)

Family members also recognized the value of the video conference system in helping to avoid hospitalization and described the NP as facilitating communication with hospital staff. This family member described how the NP had an impact on care-related expenses that the resident was responsible for, as well as costs related to staff resources in the LTC home:

They don’t have to call EMS [Emergency Medical Services] to take them to the visit or bring them home…. or the cost of a flight service or a wheelchair van… It’s not just my mom either. It’s the nursing staff…it’s the hospital at the other end with the doctors… It’s more cost effective, time effective. It’s less invasive for my mom. (S1 INT FM)

In the one exception to these findings, selected residents in one focus group expressed their preference for seeing a physician, while others remained silent in the interchange:

RES 1: I wasn’t comfortable with it [having a NP provide care].

RES 2: Neither was I.

RES 1: She’s not a doctor…I think a doctor would do more. (S3 FG RES)

One of these residents reacted negatively to the NP’s attempts to prevent her hospitalization:

Last week I was in the hospital again all night…like she [NP] wasn’t sending me there. That was me that called the ambulance…it shouldn’t be like that. (S3 FG RES)

In this case, despite the NP’s efforts, hospitalization was not avoided.

### Fostering professional working relationships

The final sub-theme involved the NP role in fostering professional working relationships. Family members and residents described the important role of the NP in collaborating and communicating with other professionals working in the LTC setting including physicians, nurses, charge nurses, and administrators, as well as professionals in hospital settings. Residents particularly focused on how the NP worked collaboratively with physicians in the homes:

She works with so many doctors here and their patients. (S2 FG RES)

NP read the reports and said…she needs the antibiotic…and she came in and spoke to me…and phoned up the doctor…I was on antibiotics that night. (S1 FG RES)

A family member described how the introduction of the NP improved the working culture for the benefit of both staff and residents:

In parachutes the NP and…they work a little better. They have a catalyst between themselves and the physicians, the other professional people…It just gets to be a better place for the residents. It gets to be a better place for the employees…The NP has made it a lot better. It runs more fluently. It’s like she is the oil to the cogs. (S2 FG FM)

Participants explained that the NP facilitated working relationships between physicians and staff:

The NP is the light switch. When you get there, it’s already on…the NP is the cushion or the catalyst between the physician and the line people. (S2 FG FM)

Family members also described how the NP established effective working relationships with professionals working in hospitals and acted as a liaison between those professionals and family members. A family member spoke about the “*networking*” between residents, family members, and other health care professionals as the “*most valuable function of the NP*” in relation to improving quality of care.

## Discussion

This study makes an important new contribution to the literature by revealing an in-depth understanding of residents’ and family members’ experiences with NPs in LTC and perceptions of what is important to them related to this role. Findings highlight that participants valued the NPs’ provision of resident and family-centred care. The sub-themes within this theme are closely aligned with some of the ‘duties’ described in McCormack’s conceptual framework of person-centredness: (a) informed flexibility or facilitating decision making through information sharing; (b) mutuality, or recognizing the others’ values as being equally important in decision making; (c) negotiation, or valuing the patient’s views and participation; and (d) sympathetic presence, an engagement that recognizes the value and uniqueness of each person [21]. Both residents and family members expressed how they valued NPs sharing information with them and involving them in decision making related to care. Their quotes also speak to mutuality of the relationship and the NP being fully present and real with residents and family members.

Study findings are also consistent with the literature on relationship-centered care (RCC). The key report titled *Health Professions Education and Relationship-centered Care* includes the statement: “Practitioners’ relationships with their patients, their patients’ communities, and other practitioners are central to health care and are the vehicle for putting into action a paradigm of health that integrates caring, healing, and community” (p.24) [42]. The current study highlights that the NP’s work is based on relationships not only with residents, but also with families and other professionals both within and outside of the LTC setting. Participants spoke eloquently about the NP role as a “catalyst”, “light switch”, and “bridge” in shaping the culture and working relationships in LTC settings. Participant quotes, such as the reference to the NP as a doula, clearly support one of the principles of RCC, namely that affect and emotion are important components of such relationships [43]. Study findings support the work of Brown Wilson who has found that relationships between staff, residents and their families are fundamental to the experience of life within the community of a LTC home; their work however did not focus specifically on the NP role [44,45].

Study results also make a contribution to our understanding of the perceptions of residents and family members about the impact of the care provided by NPs. Participants perceived that the quality of care was improved by having NPs working in LTC settings, consistent with other quantitative research [32]. Participants described improved availability of services and responsiveness to their needs, filling in gaps related to limited physician services, consistent with other research [27,29]. A strong emphasis was placed by participants on the timeliness of service provision, consistent with previous research [32]. Timeliness of response is possible because of the NP presence in the home, and the nature of relationships that are established.

The majority of participants emphasized the value of the NP in helping to avoid unnecessary hospitalization, a finding confirmed in previous quantitative research [2,13-15]. Participants emphasized how not only were cost savings important, but so too was saving residents and their usually older family members from physical and emotional strain of unnecessary trips to the hospital. The example of the NP helping to establish a videoconferencing system with the hospital was frequently mentioned as an effective strategy to help avoid such unnecessary hospital trips. However, it is clear from the negative case example cited in the findings section that the NP’s ability to prevent unnecessary hospitalization may be limited by residents choosing to go to hospital, and presumably, by family members, staff or physicians who may also prefer this outcome.

### Implications for practice, education, policy, and research

Study findings support the importance of NPs in LTC settings using person-centred and relationship-centred approaches to care. There is a wealth of recent literature that provides frameworks and strategies on how to develop such approaches [21,23,26,43,44]. NPs can act as leaders and coaches in helping staff to use such approaches in LTC settings. When there is a strong relationship between the NP and residents and families, NPs can use their expert knowledge and skill to assist residents and families to make informed choices regarding their health care, including transfers to hospital, and maintain a positive care experience.

It has been recommended that geriatric content be included in all educational programs preparing Advanced Practice Nurses such as NPs [46]. Such content is critical to fully addressing the complexity of resident health conditions such as those described in this study. It is also vital that future NPs and other health professionals receive opportunities to develop knowledge and skills in person-centred and relationship-centred care [42].

Despite research evidence on the positive resident outcomes associated with NPs in LTC settings, the total numbers of NPs in LTC remains small [46]. There is a need to explore policy and funding options for increased use of NPs in LTC. Further, there is a need to improve NP:resident ratios so that NPs have time to develop relationships with residents and families and provide quality care. In the current study, participants from the LTC setting where the NP covered two homes stated that the NP was stretched too thin and could provide better care if she was only responsible for a single home. This is consistent with a recent call for an NP in every LTC home in Ontario [6].

This study has identified the need for future research related to the NP role in LTC settings. Qualitative research on the processes and techniques that NPs use to deliver person-centred and relationship-centred care would be valuable, as well as an understanding of which processes are useful in which situations. Research on the factors influencing decisions to transfer residents to the emergency department would also be valuable.

### Limitations

A number of issues limit credibility and transferability of study results. It is possible that participants with more positive views on the role of NPs were sampled; however, interview data with residents at one site clearly reflected negative perceptions of the NP. The monetary incentive of $50 for participation may have influenced some participants to volunteer for the study. While facilitators made summary statements and asked for confirmation or clarification from participants during the focus groups and interviews, member checking where participants were contacted with summaries of findings following the initial focus group or interview was not completed.

In relation to transferability of results, there were a limited number of participants at each site, and this is in part due to the small proportion of residents who were cognitively intact and eligible to participate; nevertheless, we did find consistency of themes across settings. Only English speaking participants were included, so we did not capture the perspectives of people from different backgrounds. Extensive demographic information on the residents and family members was not collected.

## Conclusions

There is an international need to improve the quality of care provided to LTC residents and their families. The present study revealed that residents and family members in settings that had an NP viewed the NP as enhancing their quality of care. The person-centred relationships NPs develop with residents and families are a central means through which enhanced quality of care occurs. Given the small number of NPs in LTC settings, there is an opportunity for policy and decision makers at different levels of the health care system to address service inadequacies and improve the care experience through the strategic deployment of NPs in LTC settings.

## Competing interests

The authors declare that they have no competing interests.

## Authors’ contributions

JP, SK, CM, RMM, FD, NC, ESG, LSM, and KB designed the study. JP, SK, CM, RMM, FD, AWG, NC, ESG, LSM, KB, and AT participated in data analysis. All authors helped to draft the manuscript, read and approved the final manuscript.

## Pre-publication history

The pre-publication history for this paper can be accessed here:

http://www.biomedcentral.com/1472-6955/12/24/prepub
